# Mercury–carbon relationships in environmental media near artisanal gold mining sites in Guyana

**DOI:** 10.1371/journal.pone.0357021

**Published:** 2026-08-26

**Authors:** Chetwynd Osborne, D. Huy Dang, Soyini Mc Pherson, Shaun A. Watmough

**Affiliations:** 1 Environmental and Life Sciences, Trent University, Peterborough, Ontario, Canada; 2 Department of Environmental Studies, University of Guyana, Georgetown, Guyana; 3 School of the Environment, Trent University, Peterborough, Ontario, Canada; 4 Environmental Department, Guyana Geology and Mines Commission, Georgetown, Guyana; Aligarh Muslim University, INDIA

## Abstract

Elevated atmospheric mercury (Hg) concentrations from artisanal gold mining increase Hg exposure in adjacent forest canopies. Although pools and fluxes of Hg and carbon (C) within environmental media are connected, it is unclear how these Hg/C patterns may differ between areas exposed to high Hg levels and regions remote from emission sources. Here, we compare Hg/C patterns in environmental media (foliage, leaf litter, soil, water, sediment) near artisanal gold mining sites in Guyana with 25 studies worldwide to better understand Hg movement within environmental media. Patterns of Hg/C ratios in environmental media were similar between Guyana and globally synthesized studies, but Hg/C values were much higher near gold mines than the global average. Adjacent to a gold mine in Guyana, Hg/C ratios were lowest in foliage (9.8 ± 1.6 ng mg^-1^) but increased in litter (26.6 ± 3.9 ng mg^-1^) due to continued accumulation from the atmosphere. Mineral soil Hg/C ratios (5.1 ± 0.2 ng mg^-1^) are lower than litter and surface water (13.2 ± 2.5 ng mg^-1^), and stream sediment (7.3 ± 1.1 ng mg^-1^) exhibits different ratios likely reflecting a mixture of inputs from dominant flow paths (surface soils and wetlands) and erosional losses, with higher Hg/C ratios in water compared with sediment. At the global scale, peat Hg/C ratios (0.2 ± 0.2 ng mg^-1^) were nearly identical to a global synthesis of surface water (0.3 ± 0.2 ng mg^-1^), suggesting that transport of C associated with Hg from wetlands due to possible Hg accumulation from the atmosphere, is one of the primary sources of Hg to surface waters globally.

## 1. Introduction

Terrestrial ecosystems are receptors of mercury (Hg) and act as large storage pools for atmospheric Hg deposition that accumulates in surface litter and soil [1,2]. The cycles of Hg and carbon (C) are strongly linked at the landscape and biogeochemical levels, with several studies globally showing a strong linear relationship between Hg and C in soil [1,3–6]. Terrestrial vegetation is considered the missing sink in the global Hg mass balance and is estimated to absorb more than 1,000 tonnes of atmospheric Hg annually [7]. Studies have shown that atmospheric Hg is mainly absorbed by the stomata of leaves and that leaf litter constitutes 50–84% of litter biomass with higher Hg concentrations compared with other plant tissues, such as the roots and stems [8]. Therefore, fallen leaves are an important contributor to Hg fluxes to the soil [9,10].

Mercury concentrations in decomposing leaf litter are often higher than in leaves, possibly due to continued absorption of Hg from the atmosphere or transfer from soil [11]. Accumulation of Hg in surface litter alone accounts for 30–60% of total atmospheric Hg inputs to soil, which is often greater than inputs through direct wet deposition [2]. Given the affinity between organic matter (OM) and Hg in leaf litter and soil, the mobility and behaviour of Hg are also associated with the dynamics of C [1,2]. Litterfall is a major source of soil OM, with >50% of net primary production returning to the soil through plant litter decomposition. Therefore, litter decomposition is an essential step in the C cycle, and the product of decomposition normally becomes less available, thereby stabilizing soil OM and associated contaminants such as Hg [12–14].

Soil OM is the largest terrestrial C pool that accumulates through continuous plant C inputs. Carbon in soil can be transported from terrestrial ecosystems (soil, plant materials, and wetlands) to freshwater ecosystems through various flow paths [15]. The quality and quantity of C are important factors that controls the sorption of inorganic Hg and its utilization by microbial communities as an electron source in Hg methylation [16]. The anoxic conditions in sediments facilitate the biotic methylation of inorganic Hg, leading to the formation of methylmercury, the most toxic form of Hg [17,18].

Gold mining practices at the small and medium scale result in the release of high concentrations of Hg into the atmosphere, leading to its deposition into adjacent forest canopies [19]. While Hg movement and C within environmental media are connected, it is unclear how these patterns may differ in areas exposed to high levels of Hg compared with regions remote from emission sources. Relationships between Hg and C have not been extrapolated across environmental media, except for a few studies and primarily for freshwater, which report a relatively consistent value of 0.25 ± 0.20 Hg/C ng mg^-1^ in freshwaters [20–22]. However, the stoichiometry of the Hg/C relationship varies spatially in other environmental media such as plant tissues, litter, soil and sediments [1,23].

Comparing Hg/C patterns in environmental media surrounding gold mining sites with relationships observed globally could provide a greater understanding of Hg movement within environmental media. This study presents one of the first cross-media assessment of Hg/C relationships in the biosphere relative to gold mining sites in Guyana. The purpose of this study was to assess how Hg/C relationships in environmental media (foliage, leaf litter, soil, water, sediment) near artisanal gold mining sites in Guyana compare with Hg/C relationships observed in globally synthesized published studies.

## 2. Materials and methods

### 2.1. Field sampling

**Fig 1:**
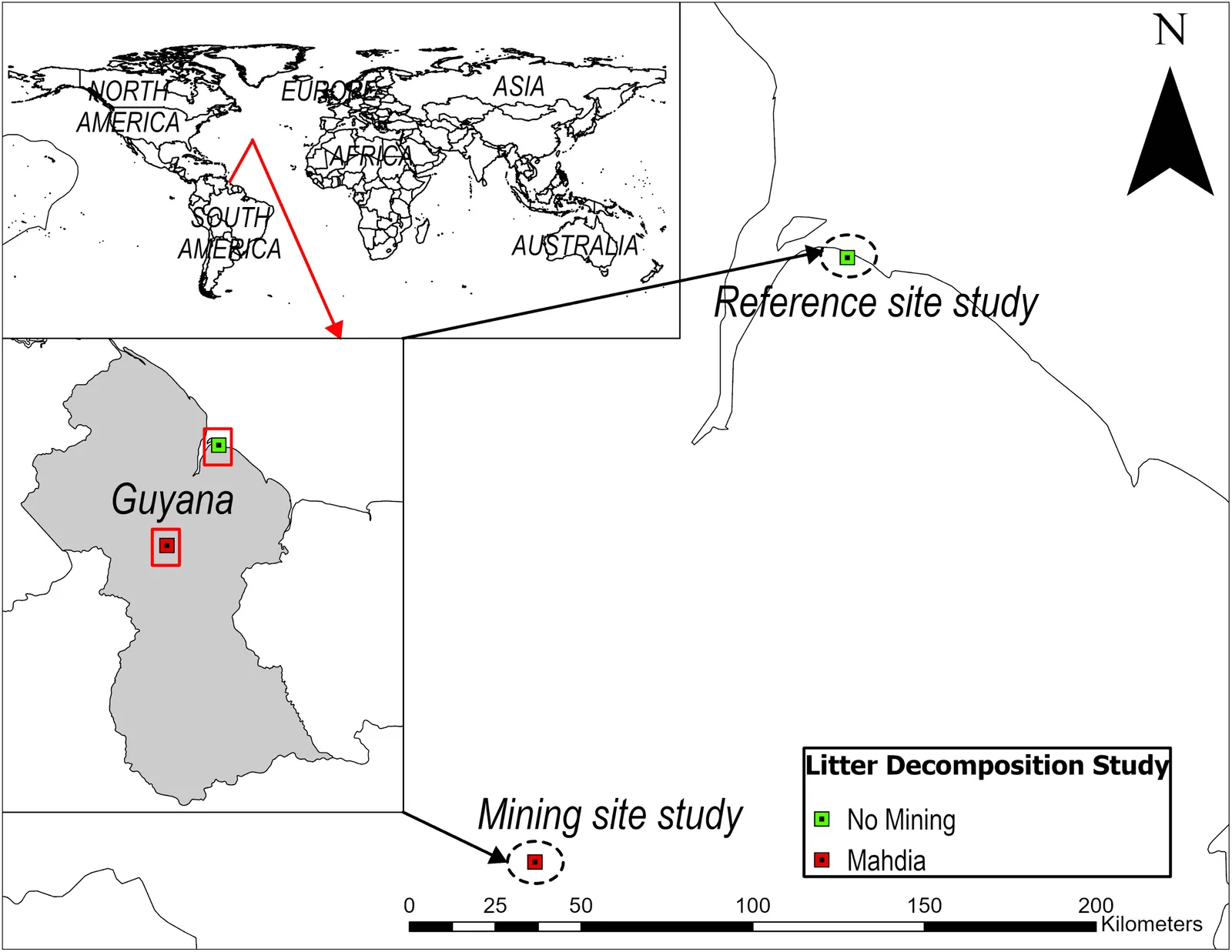
Location of deployed litterbags. Satellite imagery retrieved from Natural Earth [24].

The gold mining area was in Mahdia (mining site) (5° 16’ 54” N to 5° 17’ 3” N and 59° 3’ 32” W to 59° 5’ 46” W) (Fig 1). The mine area was separated into four zones based on spatial distance from the source of gold mining: overburden and pit (mining operation), adjacent site (1–2 km away from gold mine with forest), and distant site (2–4 km away from gold mine with forest) [25,26]. The mining site study focused on sample collection at the forested site, 2–4 km from gold mine. The Mahdia zone general wind direction is from the east, annual precipitation varies from 2,200–4,000 mm, and mean annual temperature ranges between 23–28℃ [27]. The area with no mining (reference site) was in a northeastern region of Guyana (Anna Catherina, West Coast Demerara) that is 197 km away (6° 51.991’ N and 58° 16.7381’ W) from the gold mining area in Mahdia (Fig 1). Here, the general wind direction is from the northeast, annual precipitation varies from 2,200–2,800 mm, and mean annual temperature ranges between 25–28℃ [27]. All field sampling for this study was specifically approved by the Environmental Protection Agency Board for Scientific Research on Biodiversity in Guyana under field permit numbers 20220623 BR07 and 20240507 BR007.

#### 2.1.1. Soil and sediment sampling.

To evaluate how Hg and Hg/C relationships vary in soils with distance from the gold mine in Mahdia, surface (0–10 cm) soil samples (n = 40, ca. 100 g) were collected with a stainless-steel trowel (cleaned after each sample collection to avoid cross-contamination) (July 2024) randomly within each zone (10 samples in each zone at a regular 10 m spacing). The 0–10 cm soil depth represents the primary contamination-sensitive (deposition and accumulation) and environmentally active (higher organic matter and greater microbial activity) zone of the soil profile. Similarly, sediment samples (n = 21) were collected during the dry season (July 2024) downstream (2–4 km away) of the gold mine in Mahdia to capture Hg and C values at a similar distance away from the gold mine as the litter decomposition and soil sampling sites in Mahdia. Grab samples of surface sediment (ca. 50 g) were collected in triplicate at seven (7) sample points at a depth of 0–5 cm to capture upper sediment contamination.

Collected soils and sediments were placed in labelled Ziploc® bags and stored in a cooler for transport to the University of Guyana Agriculture laboratory to be air-dried (105℃, Thermo Fisher Scientific, Model 645) [25,26].

#### 2.1.2. Soil leaching.

Soil leaching experiments [28] were done with surface soil (dried and sieved) collected from the distant site (2–4 km away from gold mine) in Mahdia (n = 5) to simulate precipitation regimes and evaluate potential Hg associated with dissolved organic carbon (DOC) mobility from soil being leached into the aquatic ecosystem. The leaching experiment used 30 mL leaching columns that mimic surface runoff mechanisms. The syringe-column leaching method was used to maintain the soil in a packed and minimally disturbed state, which allows a closer estimation of rainfall infiltration through mine-affected soils. This leaching method requires small sample mass and shorter experimental duration compared with the conventional batch or column leaching tests. Each syringe with the plunger removed was packed with 0.25 g of virgin polyester fill (avoid collection of larger soil particles with the leachate) and 10 g of soil above. The plunger was used to ensure soil was packed to a consistent density within the syringe. Approximately 40 mL of 18.2MΩ·cm water (maintain consistency in extracts) was added to each syringe with contact time that varied from 30 seconds to 180 seconds and ca. 25 mL and 15 mL leachate were collected by gravity flow only for DOC and total Hg analysis, respectively. Samples were filtered (0.45 µm filter) into 20 mL glass vials and covered with parafilm and caps until analysis [25,26].

#### 2.1.3. Preparation of litterbags and deployment.

Freshly fallen *Cecropia obtuse* leaves were collected from the mining site in Mahdia and oven dried at 35℃ to constant weight. Each litterbag (20 x 20 cm, mesh size 1 mm) received 15 g of leaf litter [25].

Litterbags (n = 12) were deployed at random points in direct contact with the soil surface in Mahdia for 14 months between June 2023 and July 2024 to determine Hg concentration in leaf litter exposed to gold mining [25,29]. Litterbags were retrieved, stored in sealed Ziploc® bags and transported to the laboratory for analysis. Litterbags were cleaned to remove any moss and soil growing on the netting and litter samples were carefully hand-cleaned to remove extraneous material.

Similarly, 12 litterbags were deployed at the reference site (no mining) for five months between July 2024 and December 2024 to distinguish soil versus atmosphere as a source of Hg to leaf litter. There was no local Hg source at the reference location in Anna Catherina (6° 51.991’ N and 58° 16.7381’ W) so atmospheric Hg is expected to be around background levels (1 ng m^-3^, global south [30]). Soil and litter samples collected from the mining location were separated into two 53 × 33 cm aluminium foil pans for the reference site experiment and each pan received replicates of six litterbags. Litterbags were harvested in December 2024 and cleaned.

Litter samples and baseline foliage (from mining and reference sites) were oven-dried at 35℃ to constant weight, weighed and ground to powder. These samples were packaged into another Ziploc® bag for storage at 4℃ to avoid any contamination until transport to Trent University Environmental Geoscience laboratory for analysis [25].

### 2.2. Foliage, leaf litter, soil, leachate, and sediment analysis

Carbon content in leaf litter and foliage was measured with a CN analyser (LECO Corporation, CN828). Dried sub-samples of foliage and leaf litter (0.08 g) were packed into tin foil cups and twist sealed to minimize headspace. Quality control of C measurements was assured by the inclusion of blanks and apple leaves certified reference materials (NIST 1515) at the start of each experimental run (30 samples) and recovery ≥ 95% was considered acceptable.

Soil and sediment organic matter (%OM) was measured by loss-on-ignition (LOI). After oven-drying (24 hours at 105℃) of soil and sediment (5 g) to constant weight, organic matter was ignited at 400℃ for 10 hours [31]. Organic matter content was converted to C using the conventional conversion factor of 0.58 [32], which can vary but these differences would have minimal impact compared to the differences among locations.

Total Hg concentration in foliage, leaf litter, soil, and sediment was measured with a Direct Mercury Analyzer (Milestone, DMA-80) by thermal decomposition, amalgamation, and atomic absorption spectrometry. Sub-samples of dried foliage, leaf litter, soil, and sediment (0.05 g) were heated to 900℃ to reduce Hg species to elemental Hg, which was loaded onto an amalgamator. Subsequent heating of the amalgamator resulted in the release of Hg vapours into a single-bean, fixed-wavelength atomic absorption spectrophotometer. Quality control of Hg measurements was assured by the inclusion of blanks and certified reference materials (NIST 1515 and *Enviro*MAT™ SS-1) at the start of each experimental run (40 samples) and recovery ≥ 90% was considered acceptable.

Dissolved organic carbon in filtered leachate samples was measured with a Total Carbon Analyzer (Shimadzu Corporation, TOC-V). Blanks and quality assurance solutions (total inorganic carbon and total organic carbon standards) were run with each batch of samples to ensure quality control. Total Hg concentration in leachate samples was measured with a Tekran 2600-CVAFS Mercury Analysis System (Tekran Instruments Corporation, Tekran 2600) by cold vapor atomic fluorescence spectrometry. A sub-sample of filtered leachate (15 mL) was added to each 40 mL glass vial, followed by 0.1 mL of 0.2 M BrCl and swirled gently to ensure complete mixing. Vials were allowed to sit overnight in the dark to form a pale-yellow solution. Calibration standards of 0 pg, 25 pg, 50 pg, 100 pg, 250 pg, 500 pg, and 1000 pg were prepared with 1 ng mL^-1^ diluted Hg standard in acid clean vials (acidified to pH < 2 with HCl and allowed to stand for a minimum of 24 hours) with clean non-talc gloves. Samples were diluted to the desired concentration, and 60 µl of 20% SnCl_2_ in 10% HCl was added to each vial (final volume of 25 mL) for analysis. Vials were capped with septa caps and shaken before loading the autosampler. Water blank (no reagent), blank reagent, and standard reference material (*Enviro*MAT™ SS-1) were run with each batch of samples to ensure quality control and recovery ≥ 95% was considered acceptable.

### 2.3. Additional data sources

The obtained C (%) values were converted to mg g^-1^ C (1 mg g^-1^ C = 0.1% C) to ultimately derive Hg/C ratios (ng mg^-1^) in environmental media (foliage, leaf litter, soil, leachate, sediment). Foliage, leaf litter, and leachate (surface runoff) samples were measured for C, while soil and sediment samples and samples from studies that reported OM or LOI data were converted based on the same conventional conversion factor of 0.58 [32]. Mercury and C values in foliage, leaf litter, mineral soil, surface runoff, and sediment for sites near the gold mine in Guyana were used to calculate mean Hg/C ratios to examine the movement of Hg through environmental media [25,26]. For comparison, Hg and C values for the same environmental media were extracted from the literature from sites with no known local Hg source to calculate Hg/C ratios to track the movement of Hg at the global scale.

Foliage, leaf litter, wetland, mineral soil (0–20 cm), river, and sediment (lake and river) Hg and C data were extracted from global studies for clean [1,4,6,11,22,33–42] and contaminated sites [33,43–49]. Data for the wetland studies were derived from 126 peat cores sampled in 16 countries across six continents (Africa, Asia, Europe, North America, Oceania, and South America) [40]. Soil Hg and C data were extracted from global studies for clean [1,3–6] and contaminated [25,26,48] sites to examine changes in the slope of the Hg/C relationship based on exposure. The validation of methodologies used to obtain Hg and C data was based on the Eurachem Guidelines and the International Union of Pure and Applied Chemistry (IUPAC) guidelines, which included characterization of selectivity, linearity, trueness, recovery, precision (repeatability and intermediate precision), limit of detection, limit of quantification, range, robustness, and measurement of uncertainty [50–52].

### 2.4. Study limitations

The absence of freeze-drying apparatus prior to the export of soil samples from Guyana resulted in the use of the air-drying approach. This drying procedure posed a limitation due to the potential loss of a small amount of Hg, as Hojdová *et al.* [53] reported that the loss of Hg in samples dried at 105℃ was 3% compared with freeze dried samples.

The absence of natural rainfall chemistry and variability in contact time for the soil leaching experiment, are potential limitations since 18.2MΩ·cm water does not fully replicate the chemical composition of natural rainfall. Also, the contact time in a syringe does not capture temporal variability of natural infiltration.

There were some inherent limitations in compiling Hg and C values from additional data sources. Heterogeneity existed among the global studies due to differences in method of analysis for Hg (sensitivity and detection limits may differ based on analytical method) and C (measurement of different fractions of carbon among studies) to derive Hg/C ratios and sampling techniques.

### 2.5 Statistical analysis

Mercury and C in mineral soil and leaf litter were subjected to Shapiro-Wilk normality test [54]. All statistical analyses (at an acceptable *α-level* of 0.05) were performed with R 4.0.4 [54] and the packages “*ggplot2*” [55], “*cowplot*” [56], and *“emmeans”* [57]. Linear regression analysis was done to evaluate the relationship between soil Hg and C at varying distances from emission source at the gold mining site in Guyana. Relationships between soil Hg and C at the global scale were compared with compiled datasets from the gold mine in Guyana. Analysis of covariance (ANCOVA) was used to compare the slopes of the regression relationships between sites. When the ANCOVA indicated significance, emtrends pairwise comparison was used to specifically compare slopes [57]. The litter decomposition experimental data were grouped according to mining site and reference site to distinguish soil versus atmosphere as a source of Hg to leaf litter. The nonparametric Kruskal-Wallis test was used to compare Hg concentration and Hg/C ratio in leaf litter. When the Kruskal-Wallis indicated significance, the Dunn test was used to determine the levels of the independent variable that differ from each other level [11,35,58]. Mean Hg/C ratios from environmental media (foliage, leaf litter, soil, surface runoff, sediment) near the gold mine were calculated and compared with mean Hg/C ratios for the same environmental media globally to provide insights into Hg movement within the biosphere.

## 3. Results

### 3.1. Variations of Hg/C relationships with distance in soil near an artisanal gold mine

**Fig 2:**
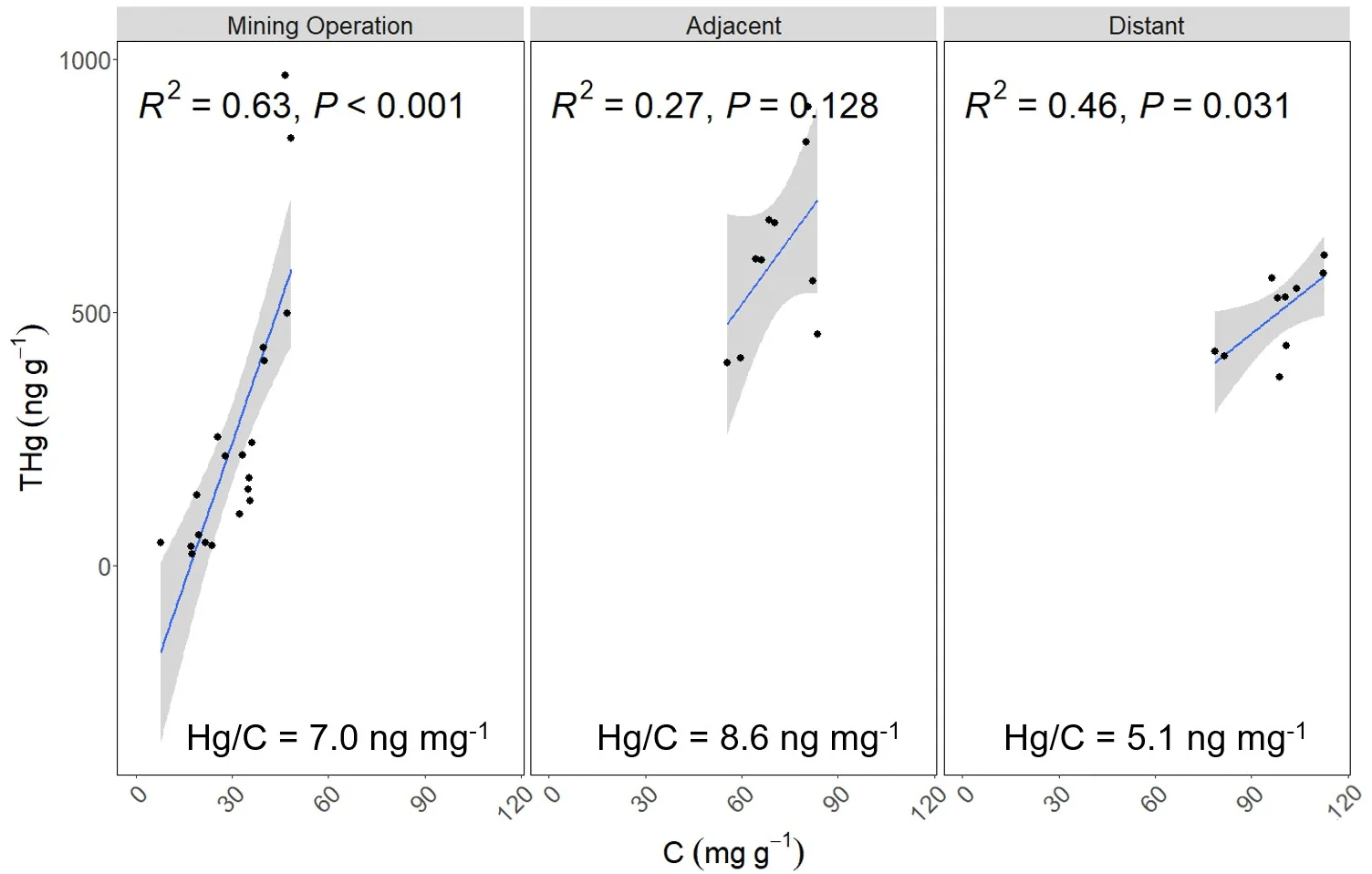
Scatter plot and linear regression between soil C and soil Hg based on distance (km) from source of gold mining in Mahdia. Adjacent = 1–2 km away from gold mine and Distant = 2–4 km away from gold mine. The grey shaded area around the solid regression line (blue) represents the 95% confidence interval.

High variability in surface soil Hg concentration was evident across the three zones, with the mining operation zone demonstrating a higher range of Hg concentrations (22.12–970 ng g^-1^) compared with the adjacent (400–906 ng g^-1^) and distant (372–614 ng g^-1^) sites (Fig 2). Soil Hg was linearly related to C, but the Hg/C ratios changed slightly and was lower at the distant site (Fig 2). The slope of these linear relationships differed significantly (*p* < 0.05, ANCOVA) between the distant site and mining operation zone compared with the other sites (Fig 2).

Mean Hg concentration was elevated in surface runoff (595 ± 37 ng L^-1^) and sediment (365 ± 88 ng g^-1^) (Table 1). Surface runoff demonstrated a higher Hg/C ratio (13.2 ± 2.5 ng mg^-1^) compared with sediment (7.3 ± 1.1 ng mg^-1^) (Table 1).

**Table 1:** Mean extracted estimates for surface runoff and sediment relative to the litter decomposition site in Mahdia (2–4 km from gold mine).

Variable	Mean ± SE
**Surface runoff (2–4 km from gold mine)**	
Hg (ng L^-1^)	595 ± 37
DOC (mg L^-1^)	49 ± 6
Hg/C (ng mg^-1^)	13.2 ± 2.5
**Downstream sediment (2–4 km from gold mine)**	
Hg (ng g^-1^)	365 ± 88
OM (%)	7.6 ± 0.6
C (%)	4.4 ± 0.3
C (mg g^-1^)	44 ± 3.3
Hg/C (ng mg^-1^)	7.3 ± 1.1

### 3.2. Leaf litter decomposition and changes in Hg and Hg/C at mining and reference sites

**Fig 3:**
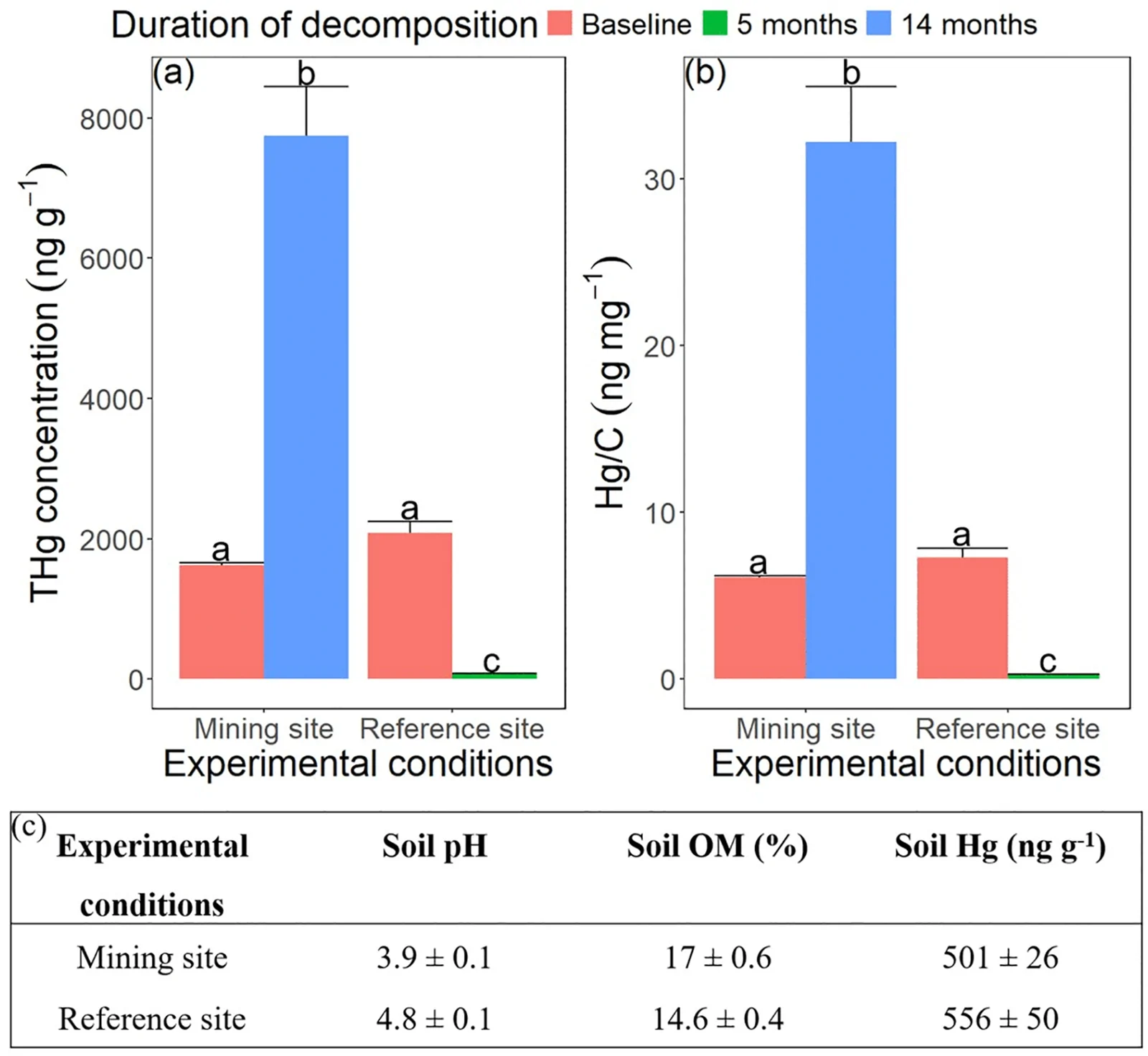
Concentrations of Hg in leaf litter after mining site (14 months) and reference site (5 months) decomposition experiments in the left panel (a) along with associated Hg/C relationships under mining and reference conditions in the right panel (b). Corresponding Hg concentration in soil underneath litter bags is also shown in the bottom panel (c). Different letters indicate statistically significant differences (p < 0.05) using Dunn’s test between each treatment per variable.

Prior to the study, leaf litter Hg and Hg/C ratios in the mining site and reference site studies were almost identical (Fig 3). However, in the mining site study Hg increased about 4-fold in leaf litter (7235 ± 819 ng g^-1^) after 14 months of decomposition compared with baseline Hg concentration (1625 ± 30 ng g^-1^) (Fig 3a). The change in Hg levels following decomposition under mining conditions also resulted in an increase in Hg/C ratio (32 ± 3 ng mg^-1^) compared with the baseline Hg/C ratio (6.1 ± 0.1 ng mg^-1^) (Fig 3b).

The reference site study was conducted using soil collected from the location of the mining site study and soil Hg concentrations at both sites were very similar ~ 500 ng g^-1^ (Fig 3c). However, in the reference site study, Hg decreased about 30-fold in leaf litter (70 ± 9 ng g^-1^) after 5 months of decomposition compared with baseline Hg concentration (2084 ± 162 ng g^-1^) (Fig 3a). This amounts to a 100-fold difference in leaf litter Hg that is decomposed in the same soil under different atmospheric Hg exposures. The change in Hg levels after 5 months of decomposition under reference conditions resulted in a large decrease in the Hg/C ratio (0.25 ± 0.03 ng mg^-1^) that was also about 100-fold lower than the mining site study (Fig 3b). The remaining leaf litter mass under reference condition was 77% of its initial mass after 5 months of decomposition, while at the mining site it was 44% of the initial mass after 14 months of decomposition, so the decrease observed at the reference site is not due to differences in leaf litter mass loss.

### 3.3. Comparison of Hg/C patterns of Guyana sites with global sites

**Fig 4:**
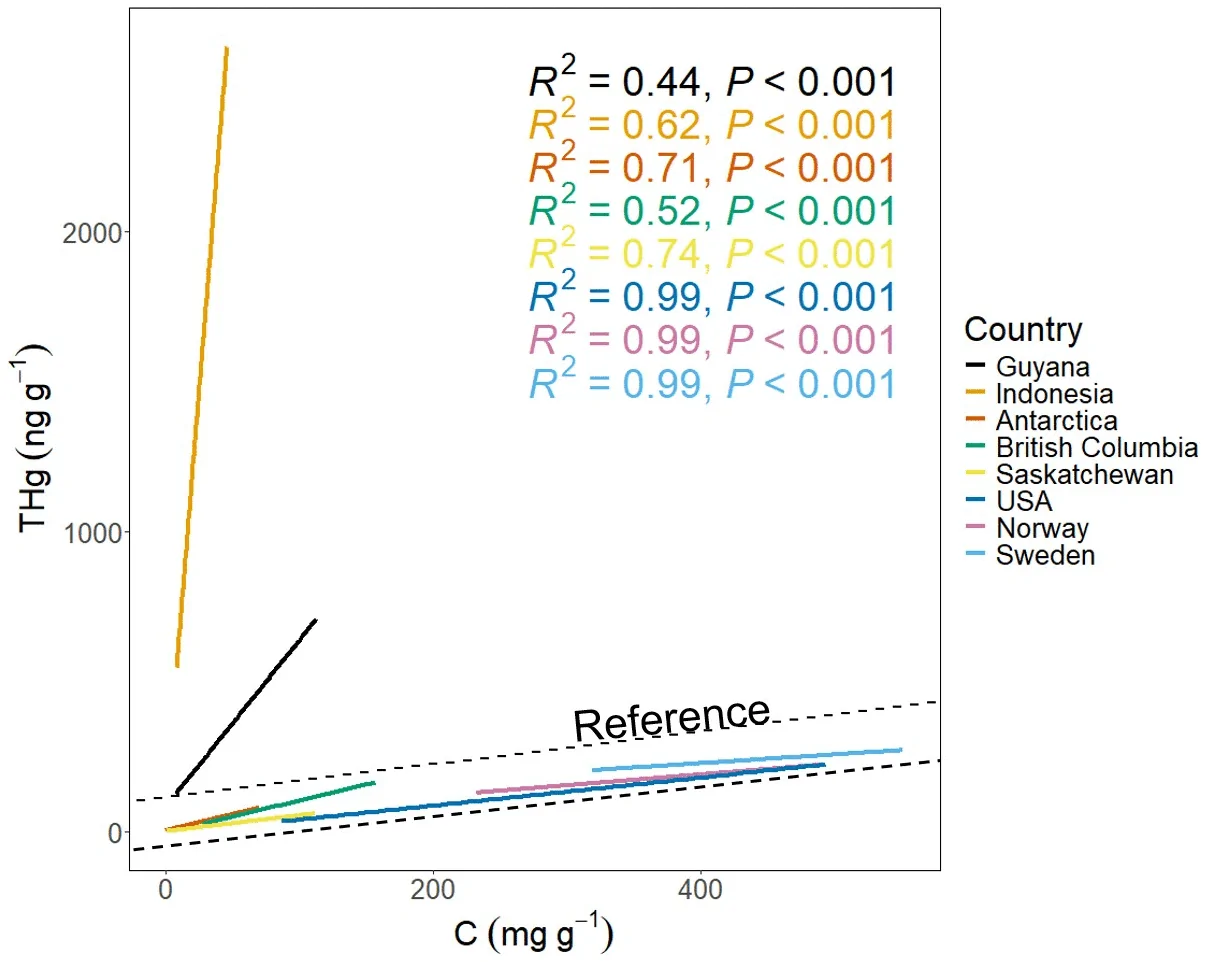
Linear regression between soil C and soil Hg – Guyana vs global studies. The area between broken lines indicates no gold mining pollution.

A strong positive relationship exists between soil C and Hg and in regions distant from point source although Hg/C are much lower (Antarctica = 4.85 ng mg^-1^, British Columbia = 1.03 ng mg^-1^, Saskatchewan = 0.82 ng mg^-1^, USA = 0.44 ng mg^-1^, Norway = 0.50 ng mg^-1^, and Sweden = 0.56 ng mg^-1^) than those observed near the artisanal gold mine in Guyana (7 ng mg^-1^) and other artisanal gold mining sites in Indonesia (63 ng mg^-1^) (Fig 4). The slope of the linear relationship near the artisanal gold mine in Guyana differed significantly (*p* < 0.05, ANCOVA) with other gold mining sites in Indonesia and distant sites from point source (Fig 4).

The Hg/C values in other environmental media (foliage, leaf litter, mineral soil, wetlands, waters, and sediment) from global studies were compiled and compared to the study site in Guyana and other contaminated sites (Table 2). At the global scale, Hg/C ratios in foliage (0.1 ± 0.2 ng mg^-1^) and soil (0.8 ± 0.4 ng mg^-1^) were much lower compared with Hg/C ratios in foliage (9.8 ± 1.6 ng mg^-1^) and soil from a gold mine in Indonesia (63 ± 7 ng mg^-1^) (Table 2).

**Table 2:** Guyana and global patterns of Hg/C (ng mg^-1^).

Hg/C ratios (ng mg^-1^)		Source [Country]
**Guyana and contaminated sites**		
Foliage (n = 2)	9.8 ± 1.6	This study [Guyana]
Foliage (n = 14, global)	14.3 ± 5.2	[19,47] [Peru, Uganda]
Leaf litter (n = 2)	26.6 ± 3.9	This study [Guyana]
Leaf litter (n = 19, global)	0.4 ± 0.1	[44,47] [French Guiana, Uganda]
Mineral soil (n = 4)	5.1 ± 0.2	This study [Guyana]
Mineral soil (n = 4, global)	63 ± 7	[48] [Indonesia]
Surface runoff (n = 1)	13.2 ± 2.5	This study [Guyana]
Sediment (n = 7)	7.3 ± 1.1	This study [Guyana]
Sediment (n = 86, global)	8.3 ± 0.8	[33,43,45,46] [India, Ghana, USA, Columbia]
**Global clean sites**		
Foliage (n = 28)	0.1 ± 0.2	[11,42] [China, USA]
Leaf litter (n = 43)	0.8 ± 0.2	[11,36,37] [Spain, USA, China]
Wetland (n = 61)	0.2 ± 0.2	[40] [global study across 16 countries]
Mineral soil (n = 420)	0.8 ± 0.4	[1,4,6,38,39] [Sweden, Norway, USA, China, Poland, Canada]
River (n = 48)	0.3 ± 0.2	[22] [global study across 14 countries]
Sediment (n = 45)	0.5 ± 0.4	[33–35,41] [India, Canada, Brazil]

n = number of sites.

**Fig 5:**
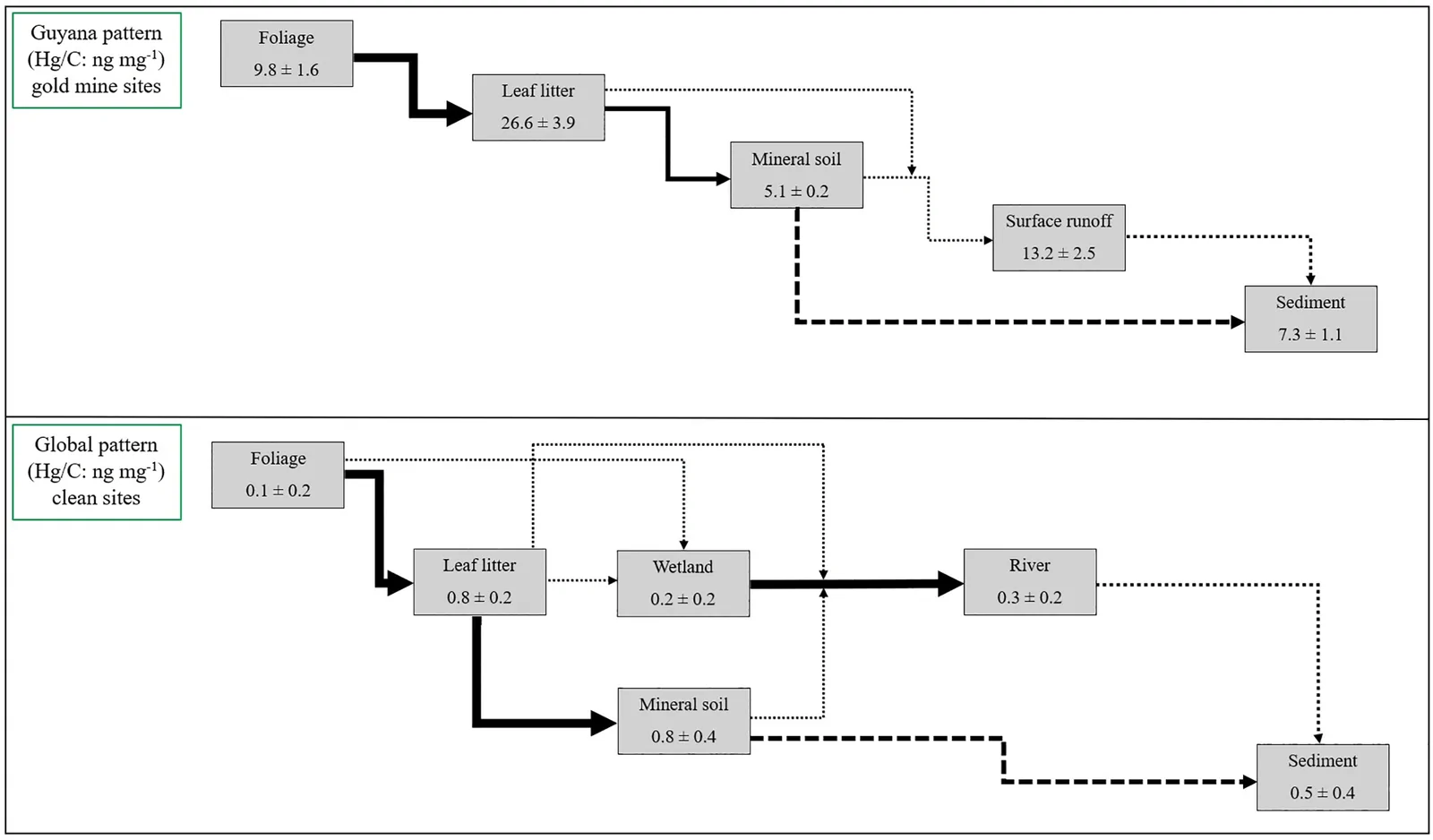
Conceptual model of Hg/C ratio in environmental media at sites near artisanal gold mining in Guyana versus global clean sites. Grey boxes indicate Hg/C (ng mg^-1^) in environmental media. Black arrows indicate the pathway of Hg and thickness of arrow indicate relative influence.

Differences in Hg/C ratios in various environmental media in Guyana exhibited similar patterns to those from globally synthesized studies, but with much higher Hg/C ratios occurring in Guyana compared with sites remote from point sources of Hg emissions (Fig 5). In Guyana, Hg/C in foliage is high (9.8 ± 1.6 ng mg^-1^) and increases in leaf litter by about 3-fold (26.6 ± 3.9 ng mg^-1^) reflecting continued accumulation of Hg in leaf litter from atmospheric exposure. In mineral soil Hg/C ratios decreased (5.1 ± 0.2 ng mg^-1^) suggesting some Hg loss relative to the source leaf litter and or inputs of C from below ground tissues (roots). The surface runoff Hg/C ratio was 13.2 ± 2.5 ng mg^-1^, which reflects interaction between water and contaminated mineral soil and leaf litter through overland flow. The Hg/C ratio in sediment was 7.3 ± 1.1 ng mg^-1^ and is similar to mineral soil suggesting erosional runoff with particulate Hg/C as a dominant contributor to Hg in sediment (Fig 5).

Collected data from 28 countries, 768 sites and 4,279 samples also showed Hg/C ratios differed among environmental media. At the global scale, Hg/C in foliage is low (0.1 ± 0.2 ng mg^-1^) [11,42] and increases in leaf litter by about 6-fold (0.8 ± 0.2 ng mg^-1^) [11,36,37] suggesting continued accumulation in leaf litter that is similar to what is occurring at the Guyana site. The mineral soil Hg/C ratio (0.8 ± 0.4 ng mg^-1^) [1,4,6,38,39] was similar to leaf litter, whereas wetland soils had much lower Hg/C ratios (0.2 ± 0.2 ng mg^-1^) [40], which are almost identical to values reported in a global synthesis of Hg/C ratios in river (0.3 ± 0.2 ng mg^-1^) [22], suggesting wetlands are one of the major contributor to Hg in freshwaters. Similar to the mine site, the Hg/C ratio in sediment (0.5 ± 0.4 ng mg^-1^) [33–35,41] falls between those observed in wetlands/surface waters and mineral soils suggesting some erosional inputs from overland flow mixing of particulate Hg/C in leaf litter and mineral soil (Fig 5).

## 4. Discussion

A strong positive relationship existed between soil C and Hg for regions close and distant from a Hg emission source, with Hg/C ratios being much lower at background sites compared with those observed near artisanal gold mines. The processes and patterns of Hg/C ratios in environmental media were similar in Guyana and globally synthesized studies, but with much higher values at contaminated sites compared with clean sites. It was evident that Hg/C ratios were lowest in foliage, with increases during decomposition due to preferential loss of some labile C relative to Hg, leading to progressive Hg enrichment in the remaining litter, along with continued accumulation of Hg from the atmosphere to decomposing litter surfaces [2,11,59]. Mineral soil Hg/C ratios were lower than ratios measured in litter, indicating some Hg loss relative to the source leaf litter and or inputs of C from below ground tissues (roots). Surface water Hg/C ratios reflected inputs from dominant flow paths (surface soils and wetlands), while sediment sample ratios were closer to mineral soils due to a greater contribution of erosional losses.

### 4.1. Relationship between Hg and C in soil near gold mines

Strong relationships between Hg and soil C were evident in Guyana although the slope of this relationship decreases slightly up to 4 km from the gold mine. Atmospheric Hg is volatilized through Hg-gold amalgamation, which is captured again by vegetation and incorporated in soil through litterfall and rainfall, where soil C binds Hg [3,6,48]. The higher slope of Hg/C observed near gold mining sites in Guyana and Indonesia suggests that Hg emissions associated with gold mining activities interact with vegetation and C, which is indicative of atmospheric Hg exposure. Close to gold mines, the periodic nature of burning gold amalgams likely results in large temporal changes in Hg/C ratios, with much higher values during a burn and much lower values occurring during intermittent periods. Gold amalgam is not always burned immediately after recovery, since miners may wait to accumulate gold over days or weeks and burn it in batches, creating intermittent emission patterns.

### 4.2. Leaf litter decomposition and relationship with Hg

In a decomposition study, leaf litter Hg collected from a forested area close to an artisanal gold mine in Guyana increased over time in the mining site but decreased in the reference site resulting in a 100-fold difference in Hg and Hg/C ratios even though decomposition took place in soil with similar Hg concentrations (~500 ng g^-1^). Therefore, accumulation of Hg from the atmosphere given the influence of gold mining activities must be the main contributor to leaf litter through wet and dry deposition. The elevated atmospheric Hg concentration (>115,187 ng m^-3^ over a 48-hr period during the burning of gold amalgam as documented by Osborne *et al.* [60]) within the gold mining environment is probably absorbed to the surface of the decaying leaf litter resulting in an increase in the litter Hg concentration [2]. Previous studies have shown that wet and dry deposition of atmospheric Hg to decaying leaf litter is an important cause of Hg increase [2,61,62]. Zhou *et al.* [63] emphasized the importance of throughfall input including gaseous oxidized Hg or particulate bound Hg to enhance increased Hg in decomposing litter. Yuan *et al.* [64] reported that the porous nature of decomposing leaf litter and the rich OM are favourable to the uptake of atmospheric Hg that is oxidized by organic S functional groups in humic substances. However, the high levels of gaseous elemental Hg emitted at the sampled gold mine in Guyana may saturate available functional groups and only a fraction of emitted gaseous elemental Hg may react. Also, some of the increase could be attributed to the rate of nutrient elements loss being much higher than the rate of Hg loss during leaf litter decomposition, which leads to Hg being concentrated in the decaying leaf litter given the distinct leaf litter mass decrease [2,62]. Studies have shown that up to 90% of the initial mass of fallen leaves is lost through decomposition due to the release of CO_2_, with another 50% of the remaining OM being lost through further decomposition under reducing conditions, predominately as CH_4_. During this process, OM also traps and accumulates deposited Hg [48,65]. Studies by Zhou *et al.* [63] and Demers *et al.* [61] reported increased concentrations of Hg and decreased litter mass following one year and two years of leaf litter decomposition, respectively. The accumulation of Hg in leaf litter on the forest floor may be a combination of new (atmospheric Hg recently emitted from anthropogenic and natural sources), recycled, and old Hg (previously deposited and bound to vegetation) [61,66].

Under reference conditions atmospheric Hg concentration is expected to be much closer to natural background levels for the global south (1 ng m^-3^) [30] given the absence of gold mines. The loss of Hg during leaf litter decomposition could be explained by microbe and OM induced mineralization of C and gaseous elemental Hg volatilization directly from litter surface [2,62,67]. Therefore, the changes in Hg/C ratio depends on if C is lost faster through mineralization or if Hg is lost faster through volatilization and alkylation followed by assimilation by microbes via microbial activities [2,62,67,68]. Obrist *et al.* [67] reported that C losses during decomposition processes may be accompanied by corresponding losses of Hg via gaseous evasion and runoff processes. The warm climate in the reference location coupled with high temperatures (25–28℃) and solar radiation could increase vapor pressure and lead to Hg loss [69].

### 4.3. Guyana versus global Hg/C patterns in environmental media

The patterns of Hg/C ratios in various environmental media were similar in Guyana and globally synthesized studies, but with much higher values at contaminated sites compared with clean sites. Therefore, the cycling of Hg and C are intrinsically linked at both contaminated sites and clean sites observed globally. In Guyana and at the global scale, Hg/C ratios were lowest in foliage, with increases during decomposition, and surface water ratios reflected inputs from dominant flow paths (surface soils and wetlands), while sediment sample ratios were closer to mineral soils due to a greater contribution of erosional losses.

Mercury is absorbed by OM given the strong sorption capacity between C and Hg [70], but this can exchange rapidly between burning and non-burning periods of gold amalgam in Guyana. Leaf litter has longer exposure time than foliage, allowing continuous Hg accumulation from the atmosphere during burning periods which increase Hg/C ratios in litter [11]. Pan *et al.* [59] reported higher Hg concentration in litter exposed to industrial zones (50 ng g^-1^) compared with areas less affected by anthropogenic activities (28 ng g^-1^). Therefore, increased atmospheric Hg exposure from contaminated sites could significantly increase the Hg/C ratio in litter. Litterfall adds Hg into soil and once buried in soil (no longer exposed to the atmosphere) belowground C inputs from fine roots lower the Hg/C ratio [1,61,71,72]. Obrist *et al.* [67] found that most of the Hg associated with soil C is not lost to the atmosphere during respiration, but it is possibly retained in the soils or subjected to other loss pathways such as runoff and mobilization. The presence of dead roots and various organic C and N rich materials released as rhizodeposition (low molecular mass and polymeric exudates, mucilage, and sloughed-off cells) from living roots contributes to higher total C supply and greater efficiency in C retention, which may lower Hg/C ratios in soil [73–75].

Mercury is transported to surface water during rain events predominantly through surface flow paths resulting in a higher Hg/C ratio. This transport mechanism is linked to the dominant influence of soil C on the enrichment behaviour and movement of Hg, leading to a significant portion of soil-bound Hg being transferred into surface water [76]. The mean Hg/C ratio in surface sediment was closer to soil than surface runoff, indicating erosion and particulate C may be a greater component. Soil erosion during rainfall-runoff events can introduce particulate bound Hg into sediment and extend the environmental risk from soils to aquatic ecosystems. During soil erosion and overland flow, surface runoff could be enriched by Hg that was deposited from the source litter [77].

At the global scale, Hg/C ratios were much lower in environmental media of these pristine sites compared with contaminated sites, but transfer of Hg through environmental media appears to operate similar to that adjacent to the artisanal gold mine in Guyana. Foliar Hg/C ratios globally were much lower than Guyana possibly due to species composition, seasonality as well as location. Han *et al.* [7] reported elevated Hg concentration for deciduous leaf species due to larger leaf area, higher number of stomata, and higher stomatal conductance compared with coniferous leaf species. These factors could enhance atmospheric Hg absorbance by leaves and dictate associated Hg/C ratios. Seasonal variation could also influence Hg/C ratios in foliage, with summer periods showing lower values compared with winter-early spring periods [78]. Here, the seasonal changes were associated with seasonal variations in tree physiology such as Hg accumulation in leaves following stomatal uptake. During winter-early spring periods water is available and there are no limitations to photosynthetic activity, so both Hg and CO_2_ diffuse through opened stomata in foliage. However, the low Hg in foliage during the summer is a possible indication of reduced Hg uptake due to reduced stomatal conductance (minimal photosynthetic activity) during these dry conditions (low precipitations) and high temperatures (> 25℃) [78]. Also, higher volatilization of Hg during hotter seasons (summer) could possibly contribute to low Hg in foliage [78]. Foliage from remote locations tends to show much lower Hg/C ratios than sites near artisanal gold mines due to the absence of Hg emission source [11,79]. Foliage is also recognized as an important interceptor of wet and dry deposition of atmospheric Hg. Foliage temporarily captures Hg during precipitation and subsequently deposit it as throughfall and litterfall (wet deposition), while in the absence of precipitation, dry deposition proceeds with the Hg deposited onto the surface of leaves [80,81]. Similar to Guyana Hg/C ratio in litter increases presumably also by continued adsorption of Hg from the atmosphere although some studies [8,61] suggest uptake of Hg from soil may be important. The accumulation of Hg in fallen litter from soil may be complexed during soil contact or translocated from soil below and likely represent old Hg fluxes [61]. Studies have shown that different leaf litter species are subject to different rates of decomposition. For instance, Hall *et al.* [72] and Ma *et al.* [11] found that deciduous leaf litter decomposed faster than coniferous leaf litter, while Demers *et al.* [61] reported the opposite. This notion could influence Hg/C ratios within litter depending on the rate of Hg inputs. Pokharel *et al.* [2] reported a significant increase in *Populus tremuloides* litter Hg concentration (>24% of original concentration) after 18 months of decomposition compared with other leaf litter species that remained close to starting levels. Here, *P. tremuloides* litter showed a lower fraction of Hg loss which is consistent with increased Hg concentration over time. Soil Hg/C values were similar to litter but there is a lot of variability likely attributable to soil depth, location, OM, and age. Mercury deposited into soil could be retained and sequestered due to the strong affinity between Hg and soil C, but evidence has shown that Hg concentration declines with soil depth along with declining soil C [67]. Morosini *et al.* [82] reported high Hg concentrations in surface soil (0–40 cm) where C content is generally higher, and Hg concentrations declined sharply with increasing depth (100 cm). Elevated atmospheric Hg concentrations near emission source may lead to an increase in Hg deposition (wet and dry deposition), which has a strong influence on distribution patterns of Hg in soil on a regional scale due to the ability of soil C to bind with Hg [11]. Interactions such as physicochemical fractioning of OM between the dissolved and adsorbed phases could account for the transport of Hg to soils [83]. The degree of decomposition is also related to C/N ratios in soil, where lower C/N ratios are generally indicative of older decomposed fractions [39]. Ma *et al.* [11] reported a significantly negative correlation between Hg/C and C/N ratios, suggesting that older and highly decomposed soils contain elevated levels of Hg.

In contrast to Guyana, wetlands are prevalent at the global scale and the mean Hg/C ratio measured in 126 peat samples from 16 countries across six continents (Africa, Asia, Europe, North America, Oceania, and South America) [40] were almost identical to the ratios obtained from an analysis of 3,578 surface water samples from 14 countries across four continents (Asia, Europe, North America, and South America) [22], suggesting that transport of C associated with Hg from wetlands is one of the primary source of Hg to surface waters globally. Mercury in wetlands could possibly be linked to atmospheric sources through precipitation as indicated by Ning *et al*. [68]. Organic rich wetland ecosystems are important sources and sinks of Hg and the transport of Hg to river is mainly driven by DOC given its affinity for Hg [1,84,85]. The coarse matrix of mineral material under the shallow riparian peat is enriched in DOC and associated Hg may be more hydrologically mobile for transport to stream channel than Hg retained in podzolized mineral soil [83]. Although wet deposition of volatilized Hg from artisanal gold mining activities serve as a pathway for localized Hg distribution, dry deposition and adsorption to organic matter (particularly through litterfall and stomatal uptake) generally contributes more to the overall distribution of Hg beyond the emission source [19]. Similar to Guyana, Hg/C ratios in global sediments were higher than surface waters and closer to soil Hg/C ratios suggesting that inputs of Hg associated with mineral soil C from erosional processes are important contributors to sediment Hg. Selvendiran *et al.* [86] reported elevated Hg concentration and DOC during high runoff periods due to wetting events, which interact with sediment. This pattern reflects DOC control on Hg due to flushing of DOC associated with elevated flow regimes. Demers *et al.* [83] also reported that the transport of legacy Hg from soils to aquatic systems possibly depends upon hydrologic flow paths and decomposition dynamics [87].

## 5. Conclusions

Organic matter return rate through litterfall depends on various factors that influence the process of decomposition and Hg movement through environmental media. A strong positive relationship exists between soil C and Hg for regions close and distant from point source, with Hg/C ratios being much lower in sites farther away from emission source compared with those observed near artisanal gold mines. The results of this study show that Hg concentration and Hg/C ratios increased in leaf litter by 100-fold for sites exposed to mining compared with sites without mining (reference site). Leaf litter with direct soil contact may function as a temporary pool for soil Hg, which may be important for risk assessments since this can aid the dispersal of contaminants through leaf litter translocation. Processes and patterns of Hg/C ratios in various environmental media were similar in Guyana and globally synthesized studies, but with much higher values at contaminated sites compared with clean sites. Here, Hg/C ratios were lowest in foliage, with increases during decomposition, and surface water ratios reflected inputs from dominant flow paths (surface soils and wetlands), while sediment sample ratios were closer to mineral soils due to a greater contribution of erosional losses. The outcomes of this study contribute to a greater understanding of the movement of Hg through environmental media. Understanding litter dynamics (decomposition) and the interaction between C and Hg provides insights into the reclamation of degraded soil since this determines the supply rate of C to soil and the reactivation of nutrient cycling. Further study should focus on the source and loss of Hg during the decomposition of leaf litter.
